# 超高效液相色谱-串联质谱法测定血浆与尿液中14种麻痹性贝类毒素

**DOI:** 10.3724/SP.J.1123.2022.05030

**Published:** 2023-03-08

**Authors:** Qiang LIN, Chao YANG, Meili LI, Jia WANG, Hanran HOU, Bing SHAO, Yumin NIU

**Affiliations:** 1.北京市延庆区疾病预防控制中心, 北京 102100; 1. Beijing Yanqing District Center for Disease Control and Prevention, Beijing 102100, China; 2.北京市疾病预防控制中心, 食物中毒诊断溯源技术北京市重点实验室, 北京 100013; 2. Beijing Municipal Center for Disease Control and Prevention, Beijing Key Laboratory of Diagnostic and Traceability Technologies for Food Poisoning, Beijing 100013, China

**Keywords:** 超高效液相色谱-串联质谱, 麻痹性贝类毒素, 血浆, 尿液, ultra-high performance liquid chromatography-tandem mass spectrometry (UHPLC-MS/MS), paralytic shellfish toxins, plasma, urine

## Abstract

人体生物基质中麻痹性贝类毒素的检测对其引起的食物中毒诊断和救治具有重要意义。研究建立了超高效液相色谱-串联质谱法测定血浆、尿液中14种麻痹性贝类毒素的分析方法。实验比较了不同固相萃取柱的影响,优化了前处理条件和色谱条件,血浆样品采用0.2 mL水、0.4 mL甲醇、0.6 mL乙腈提取后直接上机测定,尿液样品采用0.2 mL水、0.4 mL甲醇、0.6 mL乙腈提取,聚酰胺(PA)固相萃取柱净化后上机测定。采用Poroshell 120 HILIC-Z色谱柱(100 mm×2.1 mm, 2.7 μm)对14种贝类毒素进行分离,流动相为含0.1%(v/v)甲酸的5 mmoL/L甲酸铵缓冲溶液和0.1%(v/v)甲酸乙腈溶液,流速为0.50 mL/min。在电喷雾模式(ESI)下进行正负离子扫描,采用多反应监测(MRM)模式检测,外标法定量。结果表明,对于血浆和尿液样品,14种贝类毒素分别在0.24~84.06 ng/mL范围内线性关系良好,相关系数均大于0.995。尿液检测的定量限为4.80~34.40 ng/mL,血浆检测的定量限为1.68~12.04 ng/mL。尿液和血浆样品在1、2和10倍定量限加标水平下平均回收率为70.4%~123.4%,日内精密度为2.3%~19.1%,日间精密度为4.0%~16.2%。应用建立的方法对腹腔注射14种贝类毒素小鼠血浆和尿液进行测定,20份血浆样本中检出含量分别为19.40~55.60 μg/L和8.75~13.86 μg/L。该方法操作简便,样品取样量少,方法灵敏度高,适用于血浆和尿液中麻痹性贝类毒素的快速检测。

## Abstract (en)

The detection of paralytic shellfish toxins in human biological matrices is important for the diagnosis and treatment of food poisoning caused by them. An ultra-high performance liquid chromatography-tandem mass spectrometry (UHPLC-MS/MS) method was established for the determination of 14 paralytic shellfish toxins in plasma and urine. The effect of solid phase extraction (SPE) cartridges was also investigated and the pretreatment and chromatographic conditions were optimized. Under these optimal conditions, 0.2 mL water, 0.4 mL methanol, and 0.6 mL acetonitrile were successively added to plasma and urine samples for extraction. The supernatants from plasma extraction were subjected to an UHPLC-MS/MS analysis, whereas the supernatants from urine extraction were further purified using polyamide (PA) SPE cartridges and then analyzed by UHPLC-MS/MS. Chromatographic separation was conducted on a Poroshell 120 HILIC-Z column (100 mm×2.1 mm, 2.7 μm) with a flow rate of 0.5 mL/min. The mobile phase was 0.1% (v/v) formic acid aqueous solution containing 5 mmoL/L ammonium formate and acetonitrile containing 0.1% (v/v) formic acid. The analytes were detected in the multiple reaction monitoring (MRM) mode after being ionized by an electrospray ion (ESI) in positive and negative modes. Quantitation of the target compounds was performed using the external standard method. Under the optimal conditions, the method showed good linearity in the range of 0.24-84.06 μg/L, with correlation coefficients greater than 0.995. The limits of quantification (LOQs) for the plasma and urine samples were 1.68-12.04 ng/mL and 4.80-34.4 ng/mL, respectively. The average recoveries for all the compounds were 70.4%-123.4% at spiked levels of 1, 2, and 10 times the LOQs, the intra-day precisions were 2.3%-19.1% and the inter-day precisions were 5.0%-16.0%. The established method was used to determine the target compounds in the plasma and urine from mice intraperitoneally injected with 14 shellfish toxins. All 14 toxins were detected in the 20 urine and 20 plasma samples, with contents of 19.40-55.60 μg/L and 8.75-13.86 μg/L, respectively. The method is simple, sensitive, and only requires a small amount of sample. Therefore, it is highly suitable for the rapid detection of paralytic shellfish toxins in plasma and urine.

麻痹性贝类毒素(PSP)是由藻类产生的一类四氢嘌呤类化合物^[1]^,具有极性强、碱性条件下易氧化、不易被消化酶破坏等特点^[2]^。自然界中的PSP主要包括3类:(1)氨基甲酸酯类毒素,如石房蛤毒素(STX)、新石房蛤毒素等(NEO); (2)去氨甲酰基类毒素,如脱氨甲酰基石房蛤毒素(DCSTX)、脱氨甲酰基新石房蛤毒素(DCNEO)等;(3)*N*-磺酰氨甲酰基类毒素,如*N*-磺酰氨甲酰膝沟藻毒素1(GTX1)、*N*-磺酰氨甲酰膝沟藻毒素2(GTX2)等。食用被PSP污染的贝类样品后的几分钟至几个小时就会出现口腔和四肢麻木刺痛以及恶心呕吐等胃肠道症状,严重者可以危及生命。PSP的人体中毒剂量为108~900 μg STX eq/100 g,致死剂量为540~5400 μg STX eq/100 g^[2]^。GB 2733-2015《食品安全国家标准 鲜、冻动物性水产品卫生标准》中规定,PSP的限量为80 μg STX eq/100 g贝类。

PSP食物中毒很难诊断,通常是通过测定食用贝类的残留物来确定。然而在很多中毒事件中无法获得食物的残留物,且贝类样品中毒素的浓度分布不均,可能导致对毒素的严重低估或高估。通过对中毒病人血液或尿液等生物样品中毒素的检测来诊断中毒原因是一种强有力的手段。相关研究表明,PSP进入人体后在血浆^[3,4]^中主要以化合物原型存在,并以原型通过尿液^[5⇓⇓⇓⇓-10]^排出体外。液相色谱-串联质谱(LC-MS/MS)具有灵敏度高、特异性强、准确度高的优点,目前已基于LC-MS/MS建立了贝类中多种PSP同时检测的方法^[11⇓⇓-14]^。关于血液和尿液中PSP的检测方法研究目前非常有限,仅集中在STX和NEO两种毒素,尿液^[5,10]^中STX和NEO的检出限为1.00~5.10 μg/L,血浆^[3]^中NEO的检出限为0.40 μg/L,存在方法检出限高、前处理流程复杂等问题,不能满足突发事件的快速检测需求。因此,亟须建立操作简便快速、高灵敏度、多种化合物同时检测的方法,以便于快速锁定中毒因子,为临床救治提供技术支持。

本研究以14种PSP为研究对象,分别采用液液萃取和聚酰胺(PA)固相萃取柱对样品进行前处理,优化了尿液样品净化流程,有效降低了基质效应影响,结合极性色谱柱对目标物进行分离,建立了血浆和尿液中14种PSP快速、准确、高效的检测方法,为贝类食物中毒事件的快速处置提供技术支撑。

## 1 实验部分

### 1.1 仪器、试剂与材料

SCIEX Exion LC超高效液相色谱仪、SCIEX QTRAP 6500+质谱仪(美国SCIEX公司); Centrifuge 5810 R冷冻离心机(美国Eppendorf公司); Milli-Q IQ 7005超纯水器(美国Millipore公司); JXA5003 PA固相萃取柱(天津艾杰尔公司)。

甲醇、乙腈(质谱级)购自美国Sigma Aldrich公司;甲酸(优级纯)购自中国Dikma公司;14种麻痹性贝类毒素:GTX1(23.5 mg/L)、GTX2(40.6 mg/L)、膝沟藻毒素3(GTX3)(17.2 mg/L)、膝沟藻毒素4(GTX4)(7.4 mg/L)、膝沟藻毒素5(GTX5)(21.1 mg/L)、膝沟藻毒素6(GTX6)(4.94 mg/L)、脱氨甲酰基膝沟藻毒素2(DCGTX2)(35.3 mg/L)、脱氨甲酰基膝沟藻毒素3(DCGTX3) (10.4 mg/L)、*N*-磺酰氨甲酰膝沟藻毒素1(C1)(53.8 mg/L)、*N*-磺酰氨甲酰膝沟藻毒素2(C2)(16.1 mg/L)、STX(24.7 mg/L)、DCSTX(21.4 mg/L)、NEO(20.5 mg/L)、DCNEO(10.1 mg/L),均购自加拿大海洋生物科学研究所。

### 1.2 标准溶液配制

分别取1.1节中14种麻痹性贝类毒素标准溶液0.5 mL于20 mL容量瓶中,纯水定容,得到麻痹性贝类毒素混合标准中间溶液。密封储存于-18 ℃冰箱。使用时根据需要配制成不同浓度的混合标准使用溶液。

### 1.3 样品采集

由于未获得中毒病人的尿液和血浆,为获得阳性样品,本研究采用小鼠腹腔注射14种PSP后收集小鼠的血浆和尿液。吸取14种PSP混合标准中间溶液5 mL,腹腔注射4 h后使用代谢笼收集小鼠尿液,小鼠尾静脉取血收集小鼠血浆。小鼠血浆和尿液样品均保存在-80 ℃冰箱中。

### 1.4 样品前处理

#### 1.4.1 尿液样品制备

准确吸取0.2 mL尿液样品,依次加入0.2 mL水、0.4 mL甲醇和0.6 mL乙腈后涡旋混匀,12000 r/min离心5 min,吸取0.7 mL上清液,加入预先经2 mL乙腈-水(50∶50, v/v)和2 mL乙腈活化的PA固相萃取柱中,弃去流出液,加入2 mL含0.1%甲酸的乙腈-水(80∶20, v/v)溶液洗脱,收集洗脱液,过0.22 μm有机滤膜后上机检测。

#### 1.4.2 血浆样品制备

取0.2 mL血浆,依次加入0.2 mL水、0.4 mL甲醇、0.6 mL乙腈后涡旋1 min,以12000 r/min离心5 min,吸取0.4 mL上清液,过0.22 μm有机滤膜后上机检测。

### 1.5 仪器条件

#### 1.5.1 色谱条件

色谱柱:Poroshell 120 HILIC-Z色谱柱(100 mm×2.1 mm, 2.7 μm),柱温:35 ℃;流动相A:含0.1%(v/v)甲酸的5 mmoL/L甲酸铵缓冲溶液,流动相B:含0.1%(v/v)甲酸的乙腈溶液;流速:0.5 mL/min;梯度淋洗程序:0~0.5 min, 13%A; 0.5~5.5 min, 13%A~30%A; 5.5~6.0 min, 30%A~50%A; 6.0~7.5 min, 50%A; 7.5~8.0 min, 50%A ~13%A; 8.0~12.0 min, 13%A。进样体积:5 μL。

#### 1.5.2 质谱条件

电喷雾电离(ESI)源;离子源温度:550 ℃;扫描方式:正离子和负离子同时切换扫描;喷雾电压:5500 V(ESI^+^)/-4500 V(ESI^-^);气帘气压力:0.24 MPa;雾化气压力GS1和辅助加热器压力GS2:0.38 MPa。多反应监测(MRM)模式采集。其他质谱参数和保留时间见表1。

**表1:** 14种麻痹性贝类毒素的质谱参数及保留时间

Analyte	Precursor ion (m/z)	Product ions (m/z)	Collision energies/eV	Declustering potential/V	Retention time/min
Gonyautoxin 1 (GTX1)	410.1	367.1^*^, 349.1	-28, -22	-100	5.66
Gonyautoxin 2 (GTX2)	394.1	333.1^*^, 351.1	-30, -22	-130	5.74
Gonyautoxin 3 (GTX3)	396.1	298.2^*^, 315.9	29, 16	50	6.60
Gonyautoxin 4 (GTX4)	412.1	332.1^*^, 314.1	19, 27	20	6.57
Gonyautoxin 5 (GTX5)	380.2	300.2^*^, 282.2	21, 29	20	6.76
Gonyautoxin 6 (GTX6)	396.1	316.2^*^, 298.0	20, 32	30	6.94
Decarbamoyl-gonyautoxin 2 (DCGTX2)	353.3	255.1^*^, 273.0	27, 13	60	5.95
Decarbamoyl-gonyautoxin 3 (DCGTX3)	353.3	255.1^*^, 273.0	27, 13	60	6.65
N-Sulfocarbamoyl-gonyautoxin 1 (C1)	473.9	122.0^*^, 333.0	-30, -43	-40	6.60
N-Sulfocarbamoyl-gonyautoxin 2 (C2)	473.9	122.0^*^, 333.0	-30, -43	-40	6.93
Saxitoxin (STX)	300.1	204.2^*^, 138.0	30, 35	140	6.68
Decarbamoyl-saxitoxin (DCSTX)	257.1	126.3^*^, 222.3	25, 29	100	6.70
Neosaxitoxin (NEO)	316.1	220.1^*^, 126.1	34, 31	80	6.68
Decarbamoyl-neosaxitoxin (DCNEO)	273.1	225.2^*^, 126.1	31, 23	30	6.68

* Quantitative ion.

## 2 结果与讨论

### 2.1 色谱柱的选择

PSP极性较强(log *K*_ow_值处于-4.03~-1.22),使用反相色谱柱无法对其进行保留。本研究选取了Poroshell 120 HILIC-Z亲水性色谱柱(100 mm×2.1 mm, 2.7 μm),对流速、流动相以及梯度洗脱程序进行了优化,实验结果表明,按照1.5.1节色谱条件开展实验,14种PSP毒素分离效果良好(见图1),可以准确进行定性定量分析。

**图1:**
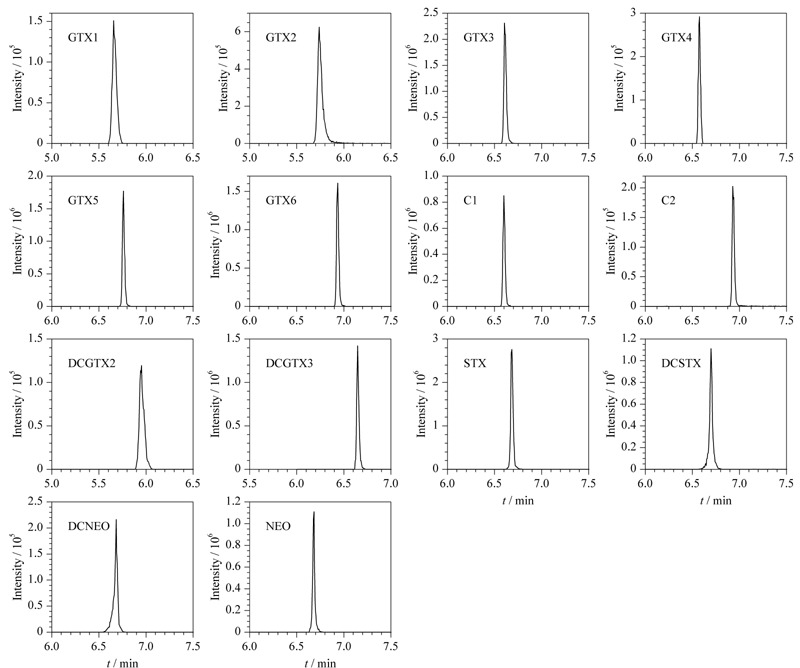
14种麻痹性贝类毒素(2.5 μg/L)的MRM色谱图

### 2.2 固相萃取柱的选择

参考文献^[7,10]^,使用0.2 mL水、0.4 mL甲醇、0.6 mL乙腈混合溶液对尿液和血浆样本进行提取,血浆样本加标回收率为71.4%~114.9%,尿液样本加标回收率为40.1%~116.7%。除回收率外,在LC-MS/MS的分析中,基质效应是评估方法的另一个重要指标。本文中基质效应以ME=空白基质样品净化提取液标准曲线斜率/溶剂标准曲线斜率×100%计算,当ME>100%,表明基质增强,ME<100%,表明基质抑制。直接采用上述溶剂提取后,血浆样本的基质效应为97%,接近100%,表明该方法对于血浆样本基质效应较小。然而尿液样本中含有极性强的盐类,直接提取会带来较强的基质效应,STX、DCSTX、NEO、DCNEO 4种化合物的基质效应在500%~810%之间,具有明显的基质增强效应,因此需要进一步使用固相萃取柱去除尿液样品中的无机盐。

石墨化炭黑固相萃取柱对于极性和非极性化合物都具有良好的亲和性,常用于贝类样品中PSP的检测^[15,16]^, PA固相萃取柱由酰胺聚合而成,其中的酰胺键可以与其他极性键基团产生氢键^[7]^,被用于尿液中STX和NEO两种毒素的检测。本研究对比了石墨化炭黑固相萃取柱和PA固相萃取柱对尿液样品提取液的净化效果(见图2)。结果表明,使用石墨化炭黑固相萃取柱对尿液样品进行净化处理,STX、DCSTX、NEO、DCNEO 4种化合物仍然表现出很强的基质效应,基质效应在450%~750%之间,且4种化合物的样品加标回收率均低于50%。采用PA固相萃取柱对尿液样品进行净化处理后,14种化合物方法的回收率在91.2%~95.0%之间,基质效应在90%~120%之间,表明该固相萃取柱对14种水溶性贝类毒素均有良好的净化作用。本研究最终选择PA固相萃取柱对尿液进行净化处理。

**图 2:**
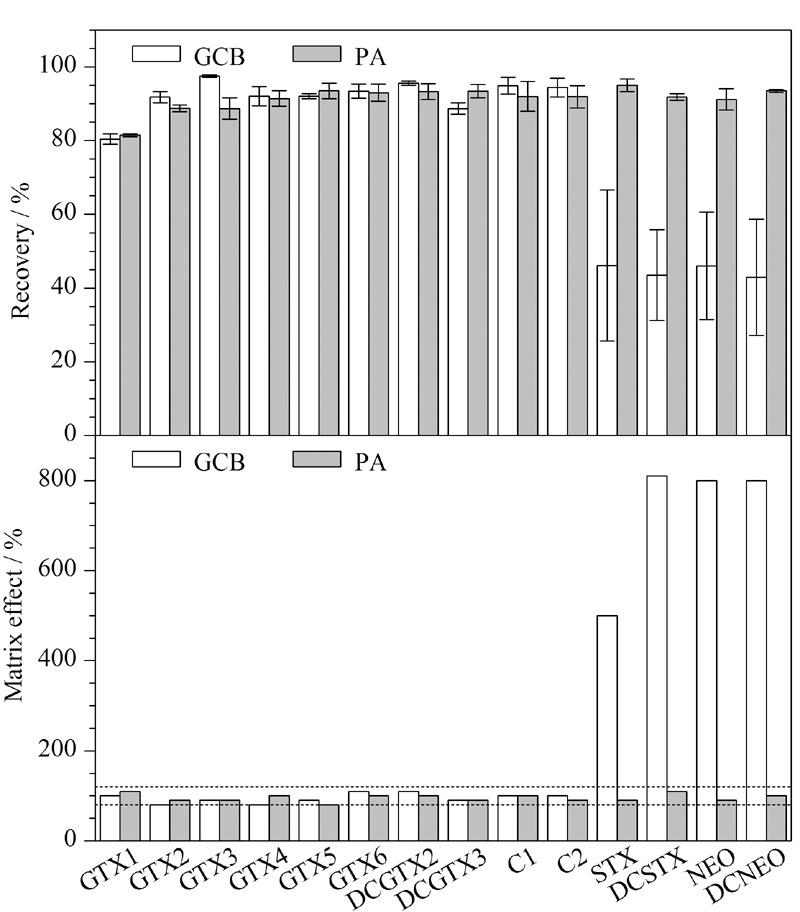
不同的固相萃取柱对尿液中14种麻痹性贝类毒素加标 回收率(*n*=6)和基质效应的影响

### 2.3 固相萃取柱净化方法的优化

在文献^[10]^基础上,本研究对洗脱液的组分进行了优化。首先,对比了含0.1%(v/v)甲酸的乙腈-水(80∶20, v/v)和乙腈-水(80∶20, v/v)两种洗脱溶液的洗脱效果。实验结果表明,使用乙腈-水(80∶20, v/v)洗脱时,14种水溶性贝类毒素加标回收率在71.2%~75.3%之间,低于使用含0.1%(v/v)甲酸的乙腈-水(80∶20, v/v)洗脱时81.1%~96.5%的加标回收率。

其次,对比了含0.1%(v/v)甲酸的乙腈与水的体积比分别为50∶50、70∶30、80∶20和90∶10时4种洗脱溶液的洗脱效果,实验结果表明(见图3),随着乙腈比例从50%升高至80%,目标物的回收率从56.1%~60.2%升高到81.1%~96.5%。当乙腈比例进一步从80%升高到90%,目标物的回收率没有进一步改善。因此,最终确定使用含0.1%(v/v)甲酸的乙腈-水(80∶20, v/v)洗脱PA固相萃取柱。

**图3:**
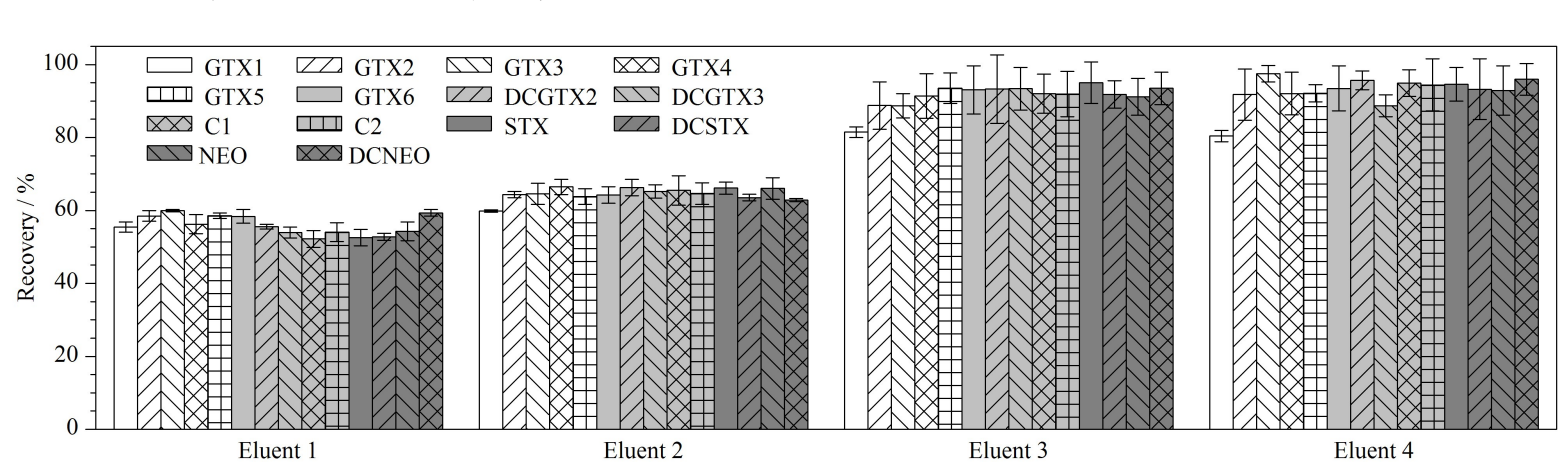
不同比例的洗脱液对尿液中14种麻痹性贝类毒素加标回收率的影响(*n*=6)

### 2.4 方法学验证

#### 2.4.1 线性范围、检出限与定量限

按照1.2节配制标准溶液,以质量浓度为横坐标,峰面积为纵坐标,绘制标准工作曲线(见表2)。结果显示,14种化合物在各自的质量浓度范围内均呈现良好的线性关系,相关系数(*r*^2^)均大于0.995。

**表2:** 14种麻痹性毒素的线性范围、相关系数、检出限和定量限

Analyte	Linear range/(μg/L)	r^2^	LODs/(μg/L)			LOQs/(μg/L)	
			Urine	Plasma		Urine	Plasma
GTX1	0.29-36.72	0.996	1.93	0.68		5.80	2.03
GTX2	0.50-63.44	0.995	3.33	1.17		10.00	3.50
GTX3	0.42-26.88	0.995	2.80	0.98		8.40	2.94
GTX4	0.36-11.56	0.996	2.40	0.84		7.20	2.52
GTX5	1.03-32.97	0.997	6.87	2.40		20.60	7.21
GTX6	0.24-7.72	0.995	1.60	0.56		4.80	1.68
DCGTX2	1.72-55.16	0.996	11.47	4.01		34.40	12.04
DCGTX3	0.51-16.25	0.997	3.33	1.17		10.00	3.50
C1	0.66-84.06	0.998	4.40	1.54		13.20	4.62
C2	0.39-25.16	0.996	2.60	0.91		7.80	2.73
STX	0.60-38.59	0.995	4.00	1.40		12.00	4.20
DCSTX	0.26-33.44	0.996	1.73	0.61		5.20	1.82
NEO	0.25-32.05	0.997	1.67	0.58		5.00	1.75
DCNEO	0.49-15.78	0.996	3.27	1.14		9.80	3.43

在空白尿液和空白血浆样品中分别进行低水平加标试验,以3倍和10倍信噪比分别确定检出限(LOD)和定量限(LOQ),具体结果见表2。尿液检测的定量限为4.80~34.40 μg/L,血浆检测的定量限为1.68~12.04 μg/L。尿液和血浆样品的定量限均优于相关检测方法^[3,5,10]^的定量限(具体数据见表3)。

**表3:** 不同检测方法对比

Analytes	Matrices	Sample volume/mL	Pre-processing method	LOQs/(μg/L)	Ref.
STX, NEO	urine	0.45	Online SPE (Oasis WCX exchange columns)	STX: 5.10, NEO: 2.62	[5]
NEO	plasma	1.00	SPE (Enviro-Clean CCX carboxylic acid cation exchange columns)	0.40	[3]
STX, NEO	urine	0.20	SPE (PA exchange columns)	STX: 1.40, NEO: 1.00	[10]
14 paralytic shellfish toxins	urine, plasma	0.20	urine: liquid-liquid extraction plasma: SPE (PA exchange columns)	urine: 4.80-34.40, plasma: 1.68-12.04	this research

#### 2.4.2 方法的准确度和精密度

分别取空白尿液和空白血浆进行加标回收试验,加标水平分别为LOQ、2倍LOQ和10倍LOQ,每个水平制备6份平行样品。以同一天6份样品平行测定结果的相对标准偏差作为日内精密度,以连续5天样品测定结果的相对标准偏差作为日间精密度。以样品的加标回收率表示方法的准确度。按照1.4节方法处理样品,方法的回收率为70.4%~123.4%,日内精密度为2.3%~19.1%,日间精密度为4.0%~16.2%(见表4),表明本方法具有良好的准确度和精密度。

**表4:** 14种麻痹性毒素的回收率和精密度(n=6)

Analyte	Average recoveries/%				Intra-day RSDs/%			Inter-day RSDs/%		
	Urine		Plasma		Urine	Plasma		Urine	Plasma	
GTX1	83.1-105.6		83.3-115.2		5.6-15.3	7.7-13.6		13.5	14.1	
GTX2	97.5-12.4		79.5-98.0		4.5-12.5	5.7-15.9		5.1	14.2	
GTX3	81.3-105.6		81.7-115.6		3.5-8.9	3.6-12.4		14.6	9.3	
GTX4	86.5-101.8		78.6-102.1		4.2-12.5	8.7-14.5		11.5	7.7	
Analyte	Average recoveries/%				Intra-day RSDs/%			Inter-day RSDs/%		
	Urine	Plasma			Urine	Plasma		Urine		Plasma
GTX5	80.6-109.3	72.5-110.4			3.3-15.6	4.5-14.7		10.3		13.2
GTX6	85.5-123.4	85.7-105.2			8.7-16.0	5.6-9.6		16.0		10.4
DCGTX2	86.7-95.8	71.5-97.6			4.3-14.0	6.3-8.9		16.2		8.3
DCGTX3	75.6-91.5	75.6-92.0			7.9-18.0	4.6-14.5		13.1		13.5
C1	70.4-93.6	71.5-94.3			14.2-18.0	6.5-11.4		10.4		15.6
C2	72.6-104.2	75.6-104.5			7.7-17.4	7.9-10.6		10.1		5.4
STX	78.9-90.4	74.6-91.0			5.5-19.1	5.8-12.4		9.2		9.4
DCSTX	77.2-112.4	77.4-87.8			5.1-14.2	3.6-17.8		8.3		11.1
NEO	76.8-114.6	71.4-114.9			5.6-12.3	5.6-12.3		9.0		15.2
DCNEO	76.5-11.3	76.9-89.7			2.3-8.9	6.2-14.7		5.0		4.0

### 2.5 样品的测定

使用本研究建立的方法检测小鼠的血浆和尿液样本,在20份小鼠尿液和血浆样本中均检测到14种麻痹性贝类毒素,含量分别为19.40~55.60 μg/L和8.75~13.86 μg/L(见表5)。

**表5:** 血浆和尿液样本中14种麻痹性毒素的浓度

Analyte	Urine/ (μg/L)	Plasma/ (μg/L)	Analyte	Urine/ (μg/L)	Plasma/ (μg/L)
GTX1	20.21	3.25	DCGTX3	35.26	5.62
GTX2	35.34	5.68	C1	41.24	10.25
GTX3	24.78	3.12	C2	26.25	15.30
GTX4	26.35	5.36	STX	30.25	10.58
GTX5	35.62	10.25	DCSTX	23.54	8.96
GTX6	19.40	8.75	NEO	24.56	10.25
DCGTX2	55.60	13.86	DCNEO	36.78	15.56

## 3 结论

本研究对血浆、尿液的前处理条件进行了优化,血浆经过简单的液液萃取,尿液经过优化的固相萃取流程,即可上机检测14种麻痹性贝类毒素化合物。该研究建立的方法具有目标化合物种类多、操作简便、灵敏度高的特点,适合发生食物中毒时快速开展尿液、血浆中多种毒素的筛查、定量工作。
